# Health workers labor market before and during the Covid-19 pandemic: Health sector capacity of Serbia

**DOI:** 10.1093/eurpub/ckac131.283

**Published:** 2022-10-25

**Authors:** M Šantrić Milićević, S Mandić-Rajčević, A Stevanovic

**Affiliations:** 1 School of Public Health and Health Management, Faculty of Medicine, University of Belgrade, Belgrade, Serbia; 2 Institute of Social Medicine, Faculty of Medicine, University of Belgrade, Belgrade, Serbia

## Abstract

**Background:**

During the COVID-19 pandemic surges, healthcare stakeholders were concerned with the sufficiency of available health workforce capacity. In this study we examined the changes in the supply and demand of physicians, nurses and care workers in Serbia over the period 2011-2021.

**Methods:**

The National Employment Service (NES) data on total number of unemployed physicians, nurses and care workers, and vacancy data in health sector were described using the annualized % change for the period 2011-2021. The long-term duration of unemployed female physicians and nurses was further analyzed.

**Results:**

In 2021, NES has registered total of 13,332 unemployed physicians, nurses and care workers, out of which the majority were females (79%), and nurses and care workers (88%). 2021 data on vacancies showed that only 16% of unemployed workers were needed. The peak of health workers unemployment was in 2016, highlighting the period of unemployment rise (2011-2016) at an annualized rate of 3.7% for medical doctors’ specialists, 6.4% for medical doctors without specialization, and 3.2% for nurses and care workers, and the period of unemployment decline (2017-2021) at an annualized rate of -7.9% for medical doctors’ specialists, -10.9% for medical doctors without specialization, and -5.9% for nurses and care workers. The annualized rate of decline was the lowest for female nurses and care workers. On average 53% of all long term unemployed medical doctors, nurses and care workers were women. In comparison to 2019, during the COVID-19 epidemic in 2020 and 2021 the number of vacancies for specialists and nurses and care workers has increased by one-third.

**Conclusions:**

The study indicated a continuous mismatch between the supply and demand of physicians and nurses in Serbia (a surplus of some categories of nurse-specialists versus a shortage of some doctor specialists). The Serbian stakeholders need to urgently intervene regarding the long-term unemployment of female health workers.

**Key messages:**

• NES data imply a low capacity of the Serbian health sector to absorb the huge numbers of (long-term) unemployed health workers.

• It is necessary to thoroughly examine and counteract the causes of the dramatic number of unemployed health workers on the NES records in Serbia.

